# Next Generation Sequencing Reveals the Hidden Diversity of Zooplankton Assemblages 

**DOI:** 10.1371/journal.pone.0081327

**Published:** 2013-11-07

**Authors:** Penelope K. Lindeque, Helen E. Parry, Rachel A. Harmer, Paul J. Somerfield, Angus Atkinson

**Affiliations:** Marine Life Support Systems, Plymouth Marine Laboratory, Plymouth, Devon, United Kingdom; Stazione Zoologica, Italy

## Abstract

**Background:**

Zooplankton play an important role in our oceans, in biogeochemical cycling and providing a food source for commercially important fish larvae. However, difficulties in correctly identifying zooplankton hinder our understanding of their roles in marine ecosystem functioning, and can prevent detection of long term changes in their community structure. The advent of massively parallel next generation sequencing technology allows DNA sequence data to be recovered directly from whole community samples. Here we assess the ability of such sequencing to quantify richness and diversity of a mixed zooplankton assemblage from a productive time series site in the Western English Channel.

**Methodology/Principle Findings:**

Plankton net hauls (200 µm) were taken at the Western Channel Observatory station L4 in September 2010 and January 2011. These samples were analysed by microscopy and metagenetic analysis of the 18S nuclear small subunit ribosomal RNA gene using the 454 pyrosequencing platform. Following quality control a total of 419,041 sequences were obtained for all samples. The sequences clustered into 205 operational taxonomic units using a 97% similarity cut-off. Allocation of taxonomy by comparison with the National Centre for Biotechnology Information database identified 135 OTUs to species level, 11 to genus level and 1 to order, <2.5% of sequences were classified as unknowns. By comparison a skilled microscopic analyst was able to routinely enumerate only 58 taxonomic groups.

**Conclusions:**

Metagenetics reveals a previously hidden taxonomic richness, especially for Copepoda and hard-to-identify meroplankton such as Bivalvia, Gastropoda and Polychaeta. It also reveals rare species and parasites. We conclude that Next Generation Sequencing of 18S amplicons is a powerful tool for elucidating the true diversity and species richness of zooplankton communities. While this approach allows for broad diversity assessments of plankton it may become increasingly attractive in future if sequence reference libraries of accurately identified individuals are better populated.

## Introduction

The seas and oceans cover >70% of the earth’s surface and contain exceptionally diverse fauna. The pelagic fauna are dominated, in terms of abundance and biomass, by plankton. The importance of these plankton for marine ecosystem functioning is widely recognized and includes their role in biogeochemical cycles [1] and the transfer of energy from primary producers to higher trophic levels, such as commercially important fish [2]. Within the plankton, holoplankton are permanent residents (e.g. most copepods, salps and chaetognaths) while meroplankton are only planktonic for part of their lives, usually the larval stage (e.g. benthic species and fish larvae). 

Globally, there is an increasing need to measure marine biodiversity and to quantify the rate at which it is changing [3]. It is therefore critical to study zooplankton community structure and diversity, and to monitor its response to environmental change [4,5]. Despite its importance, understanding of zooplankton biodiversity is hindered by a persistent problem of correctly identifying the species present [6]. The taxonomy and identification of marine zooplankton has traditionally been based on morphological characteristics using a light microscope [7]. However, owing to the similar morphologies and restricted diagnostic features of zooplankton, positive identification is frequently complicated, challenging and time consuming [8]. At present most juvenile instars can be morphologically identified with ease to only Phylum or Class, and rarely to Order or Family [9]. 

In such cases it is possible to use molecular techniques to improve the resolution of identification. Over the past 20 years work has been undertaken to utilise DNA sequences of homologous gene regions to design molecular techniques, such as RFLP [10-12] and multiplexed species-specific PCR [13,14] to discriminate between zooplankton species at any developmental stage. These techniques can distinguish between the different species of a particular genus but cannot be scaled up to identify all the different species in a mixed assemblage. 

Extraction of DNA from whole community samples, amplicon generation of a suitable gene fragment and subsequent sequencing would enable studies of whole ecosystem diversity. Zooplankton diversity has been analysed through single-gene sequencing of a community sample by amplicon generation, construction of a cDNA library and subsequent sequencing using traditional Sanger methods [15]. However, such environmental clone-library surveys yield only a modest number of sequences with which to evaluate taxon richness, so are inadequate for processing complex environmental samples, especially for large-scale studies [16]. In 2005, massively parallel next generation sequencing (NGS) platforms became widely available [17], bringing with them the technology to recover DNA sequence data directly from whole community samples from a variety of ecosystems including freshwater, marine, soil, terrestrial and gut microbiota [16] in a feasible and affordable manner. 

NGS platforms have now been used for a broad range of applications to address diverse biological problems [18]. Most NGS studies in the marine environment have investigated bacteria and archaea diversity [19-24] with eukaryotic environmental metagenetics being a relatively newly emerging field [25]. Amplicons of 18S rDNA have been utilised to analyse marine eukaryotic microbiota [26], metazoa [27], meiofauna [25,28], fish [29] and the diet of lobster larvae [30], but to date limited NGS studies have been undertaken on marine plankton communities [31]. 

Regardless of the ecosystem studied, the vast majority of environmental studies using NGS have employed the 454 pyrosequencing platform, mainly because of its longer sequence read lengths compared to other technologies [16]. As whole ecosystem studies will contain novel genomes that have yet to be fully mapped, the longer reads afforded by the 454 platform increase the chance of assigning taxonomy to the sequence using a reference database such as NCBI or GenBank.

Since 1988, a plankton time series has been maintained at the monitoring station L4 as part of the Western Channel Observatory off Plymouth, UK. Station L4 is situated about 13 km south of Plymouth with an average water depth of 54 m. It is in a region with both cold temperate and warm temperate species [32] and is influenced by seasonally stratified and transitional-mixed waters [33]. L4 is a well-studied site at which an impressive suite of environmental and biological characteristics have been recorded for over 20 years [34,35], including weekly analysis of zooplankton community structure and abundance using microscopic identification [5,36]. Given the wealth of knowledge associated with the site, Station L4 is an ideal location to trial NGS of a mixed zooplankton assemblage. 

This paper aims to compare and contrast the taxon richness and diversity of whole community zooplankton samples derived from traditional microscope-based morphological analysis with the metagenetic analysis of homologous 18S rRNA genes using the 454 pyrosequencing platform. The differences, strengths and weaknesses between the two approaches are considered.

## Materials and Methods

### Ethics Statement

This manuscript details results obtained from a field study, namely the collection of plankton samples from the Western English Channel Observatory long-term monitoring station L4 (50° 15'N, 4° 13'W; depth 50m) in September 2010 and January 2011. As specified by the Marine Management Organisation (MMO) no specific permissions were required for the collection of water samples used in this study as the site used is not subject to any relevant conservation or protection legislation. The field studies did not involve endangered or protected species. 

The organisms in the plankton sample do not require ethics approval by a specific committee as they are unregulated animals. However, every effort was taken to ameliorate animal suffering; following collection, samples were returned immediately to the laboratory and processed as detailed in the ICES Zooplankton methodology manual [37]. 

### Sample collection

Sampling was carried out on 27^th^ September 2010 and 25^th^ January 2011 at the Western Channel Observatory (www.westernchannelobservatory.org.uk) long-time series station L4 (50° 15.00’N, 4° 13.02’W). Four replicate vertical hauls were performed on each date with a 200 µm mesh WP2 plankton net from 50 m to the surface. In September 2010 two of the replicate hauls were preserved in 4% buffered formalin for assessment using a light microscope and two were concentrated by centrifugation, and excess seawater removed prior to being frozen at -20°C in 50 ml falcon tubes for downstream molecular processing. In January 2011, half of each of the four replicate hauls was preserved in formalin for microscope analysis and the remaining half of each sample was treated for molecular processing as described above. 

### Molecular identification

#### DNA extraction

Samples were prepared for genomic DNA extraction by thawing prior to centrifugation at 3200 *g* for 4 minutes to pellet the zooplankton. Excess seawater was removed and 15 ml homogenizing solution (400 mM Tris-HCl pH 8, 60 mM EDTA, 105 mM NaCl, 1% SDS, 0.28 µg/µl RNase) added prior to physical homogenisation, using a 10 ml syringe and 19 G needle, and incubation at 37°C for 30 minutes. After this 250 µg/ml proteinase K was added and the samples were incubated for a further 30 minutes at 37°C. 4.28 ml Sodium perchlorate (5M NaClO_4_) was added and the samples were shaken at room temperature for 20 minutes. They were then physically homogenised as before and incubated at 65°C for a further 20 minutes. The homogenate was extracted once with phenol/chloroform pH 8.0, once with chloroform and precipitated with 2.5 volumes of 100% ethanol at -80°C for 1 hour. The samples were washed with 70% ethanol, air dried overnight, then re-suspended in 1.5 ml DNA-grade water (Fisher Scientific) and left at 55°C for 30 minutes then at room temperature for 3.5 hours. The DNA extractions were analysed to assess quality and quantity of DNA present using a NanoDrop 1000 Spectrophotometer (ThermoScientific, Delaware USA). 

#### 454 sequencing

Primers (SSU_F04 and SSU_R22), designed by Fonseca et al [27], were chosen for amplicon generation. These primers target a homologous region of the 18S nuclear small subunit (nSSU) ribosomal RNA (rRNA) gene and flank a region that is highly divergent. Fusion primers were developed to include a proprietary adaptor sequence and a four nucleotide key tag. PCR amplification was performed in triplicate using 1 µl of genomic DNA template (1:10 dilution) in 25 µl reactions containing 5 µl of 5x buffer, 2.5 µl 2 mM dNTPs, 2 µl 25 mM MgCl_2_, 9.25 µl DNA-grade water (Fisher Scientific), 2.5 µl of primers (10 µM) and 0.25 µl of GoTaq Flexi (Promega). The PCR conditions involved a 2 min denaturation at 95°C followed by 30 cycles of 1 min at 95°C, 45 s at 55°C, 1 min at 72°C and a final extension of 10 min at 72°C. No template controls were included. Electrophoresis of pooled triplicate PCR products and negative controls was undertaken on a 1% agarose gel and the 450 bp amplicons were extracted using the QIAquick Gel Extraction Kit (Qiagen). The amplicons were purified using Agencourt AMPure beads (Beckman Coulter) following the manufacturer’s instructions. The amplicons were quantified using Quant-iT PicoGreen dsDNA assay kit (Invitrogen) and then diluted to stocks of 1 x 10^9^ molecules µl^-1^ required for the emPCR.

Amplicons from all six samples (2 in September and 4 in January) were sequenced on a Roche 454 FLX platform. Technical replicates were also performed on one sample from September (haul 2) and one sample from January (haul 4) to determine the effects of increasing the depth of sequencing. DNA sequencing was carried out using a GS FLX Titanium pyrosequencer (Roche) using Titanium chemistry. The fragments in the amplicon libraries were bound to DNA capture beads under conditions recommended by Roche (emPCR Method Manual – Lib-L, revised Jan 2010) to ensure only one fragment per bead. The beads were emulsified and a PCR was performed. Following the Roche Sequencing Method Manual (November 2010) the beads were then recovered, washed and enriched before being loaded onto a PicoTiter-Plate fitted with an 8 region bead loading gasket and sequenced unidirectionally.

#### Sequence data processing

The 454 sequencing reads were processed using the Qiime (Quantitative Insights into Microbial Ecology, v1.3.0) pipeline [38] as flowgrams (.sff files). The data was processed following the steps recommended in the Qiime processing 18S data tutorial (http://www.qiime.org/tutorials/processing_18S_data.html). Default settings were used for all parameters, namely an operational taxonomic unit (OTU) threshold of 0.97, 0 primer mismatches, 0 ambiguous bases, a maximum length of homopolymer run of 6, 200 nucleotides as a minimum sequence length and 1000 as a maximum sequence length. The samples were not multiplexed so the barcode area of the mapping file was left blank and the split libraries script was altered accordingly. The data were then de-noised using the de-noiser wrapper within Qiime to remove the sequence errors characteristic of 454 sequencing machines [39]. Chimeras were identified using ChimeraSlayer [40] and rejected from the dataset before construction of the OTU table. The OTUs were assigned using UCLUST [41], a de novo OTU picker within Qiime, a representative set of sequences was then generated and these sequences were assigned taxonomy using the SILVA release 108 database [42]. Additionally, due to the relatively low numbers of OTUs (205), the representative sequences were also manually assigned taxonomy using the BLASTN search of the NCBI non-redundant dataset.

### Morphological identification

Formalin-preserved samples were examined by microscopy to identify the zooplankton community morphologically. Samples were analysed following the protocol used routinely for the zooplankton time series at L4 [5,43], except that rather than two separate net samples each being examined and the results averaged, here we enumerated 4 half-samples in Jan 2011 and 2 samples as normal in Sept 2010. Due to the high number of organisms in the samples it was necessary to take two sub-samples to make counting feasible. This represents normal practice when enumerating zooplankton samples by microscopy [44]. Firstly, each sample was poured into a 63 µm sieve, washed into a round-bottomed flask and made up to 300 ml with tap-water. An aliquot of either 2.5 or 5 ml was taken from the flask with a Stempel pipette, aiming to collect at least 200 organisms for identification. All organisms in this sub-sample were identified and counted using an Olympus SZX16 microscope. A second sub-sample of ^1^/_4_ or ^1^/_8_ of the full sample was then examined to identify and enumerate larger and rare organisms that were not present in the small sub-sample.  Generally organisms were identified to species or genus level where possible, but some groups, in particular meroplanktonic larvae (e.g. Polychaeta, Cirripedia, and fish larvae) could only be identified to major taxonomic groups. Copepods, which generally dominate plankton samples at L4, were identified to genus or species, with the most common calanoids also distinguished to stage level (male/female/juveniles). The copepodites of some small calanoid genera (*Pseudocalanus*, *Ctenocalanus* and *Clausocalanus*) are difficult to identify to genus routinely and so were counted as “calanoid juveniles”.

Zooplankton abundances (no. of individuals m^-3^) were calculated using net mouth area and haul depth to estimate the volume of water filtered by the net, assuming that filtration was 95% efficient. 

### Assessment of morphological and metagenetic diversity

OTU tables generated by Qiime and abundance data from morphological analysis were analysed using the PRIMER version 6 software package [45]. Biodiversity measures were calculated for replicate samples. Differences in community structure between groups of samples were explored using Bray-Curtis similarities calculated from square-root transformed abundances. Similarity Profiles analysis [46] was used to test for multivariate structure. Rarefaction curves were constructed based on randomly permuted orders of samples. Where appropriate, the significance of differences in numerical values, such as diversity measures and counts, among groups of samples was tested using Student’s t-test.

## Results

### Assignment of Molecular Operational Taxonomic Units (OTUs)

Concentrations of DNA in the sample extracts from the 6 plankton hauls (September and January) ranged from 94 to 1128 ng µl^-1^. Amplicons of the 18S small subunit rRNA gene were generated for each sample and these amplicons demonstrated good levels of DNA, ranging from 5.3 to 29.1 ng µl^-1^. Amplicons from all six samples, plus two technical replicates of samples 2 and 4 which were sequenced in duplicate, generated a total of 485,817 DNA sequences. The raw reads from the 454 sequencing are publically available in the European Nucleotide Archive (http://www.ebi.ac.uk/ena/data/view/PRJEB4768). Following quality control and removal of chimeras 419,041 sequences were left, representing a 13.7% loss of sequences (Table 1). The average read length of the sequence fragments was 399 bp. These sequences were clustered into a total of 205 OTUs using a 97% similarity cut-off [47]. Using the SILVA 108 database about 65% of sequences were assigned to an “unknown” category (OTUs that are < 97% homologous to any other sequence in the database). Due to these high levels of ‘unknowns’ when using the SILVA 108 database a representative sequence from each OTU was also used in a BLASTN search against the NCBI non-redundant nucleotide dataset, using the criteria that the BLASTN coverage score was over 90 and the BLASTN homology was greater than 97%. The species with the highest score in the BLASTN result list was assigned to the OTU. If more than one species showed >97% similarity to an OTU, then it was assigned to the common genus or the lowest shared taxonomic level. This resulted in reducing the number of ‘unknowns’ to 2% of sequences. The allocation of taxonomy using BLASTN search against the NCBI dataset identified 135 OTUs to species level, 11 to genus level and 1 to order [48]. There were a further 58 OTUs that could not be identified as they did not meet our BLASTN criteria, and were therefore classified as unknowns. These 58 OTUs (sequences = 8800) account for less than 2.5% of the sequences from the September hauls and less than 1.8% in the January hauls. In all subsequent data analysis the OTUs are referred to using their BLAST-assigned taxonomy. 

**Table 1:** Sequence loss resulting from Qiime quality control.

	**SEPTEMBER 2010**			**JANUARY 2011**					
**Sample**	**1**	**2**	**2a**	**3**	**4**	**4a**	**5**	**6**	**TOTAL**
**Accession #**	ERS 360365	ERS 360366	ERS 360371	ERS 360367	ERS 360368	ERS 360372	ERS 360369	ERS 360370	
**# Raw reads**	89167	55961	22778	112114	25030	22789	59432	98546	485817
**# Sequences after de-noising and chimera removal**	81286	50377	20543	104163	21826	19979	52796	68071	419041
**Sequences lost (%)**	8.8	10.0	9.8	7.1	12.8	12.3	11.2	30.9	

Several of the assigned sequences showed very high degrees of similarity to common locally recorded taxa, with a number of OTUs showing 100% homology to the NCBI reference sequence. These sequences include those from a diverse range of taxa including arthropods, bivalves and gastropods, and include species that are commonly documented at L4 such as *Calanus helgolandicus.*

### Assignment of Morphological Operational Taxonomic Units

The assignment of OTUs for samples identified morphologically was based on the highest level of taxonomic resolution possible by the analyst. A total of 2058 organisms were counted and 58 OTUs were recorded, with 4 being identified to phyla, 9 to class, 5 to order, 2 to family, 8 to genus and 30 to species level [43]. Within many of the copepod OTUs, sex and developmental stage information was also recorded. For example *Temora longicornis* is recorded as one OTU however, within this OTU, morphological analysis is able to give information on the number of juveniles, males and females in the plankton haul. In contrast, morphological identification of copepod nauplii can only be taken to subclass level.

### Replicate analysis and sequence coverage

Based on square-root transformed abundances, the average Bray-Curtis similarity among samples was 75% in September and 82% in January. SIMPROF showed significant differences in community structure between the two sampling times, but no evidence of any differences among samples within times. 

**Figure 1:**
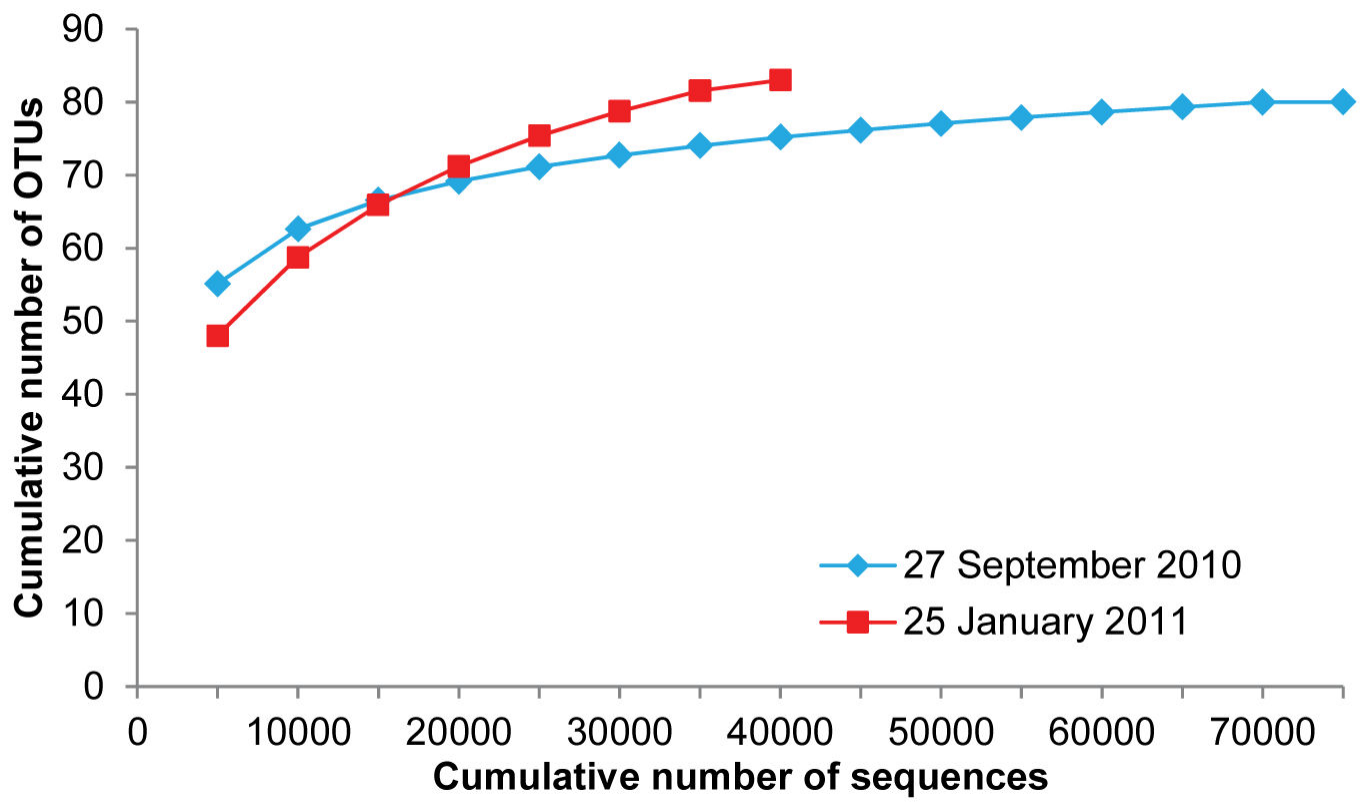
Rarefaction curve of repeat samples in September 2010 and January 2011. Rarefaction analysis for the 112725 18S small subunit gene sequences from repeat samples in September 2010 (2 and 2a) and January 2011 (4 and 4a).

As part of the metagenetic analysis, technical sequencing replication was performed on samples 2 and 4 (sample replicates = 2a and 4a) to determine whether increased sequence coverage contributed any further OTUs. The rarefaction curve (Figure 1), which plots the cumulative number of sequences generated by the original and replicated sequencing runs against the number of OTUs, appears to be nearing asymptotic values. 

### Comparison of metagenetic and morphological taxon richness estimates

**Figure 2:**
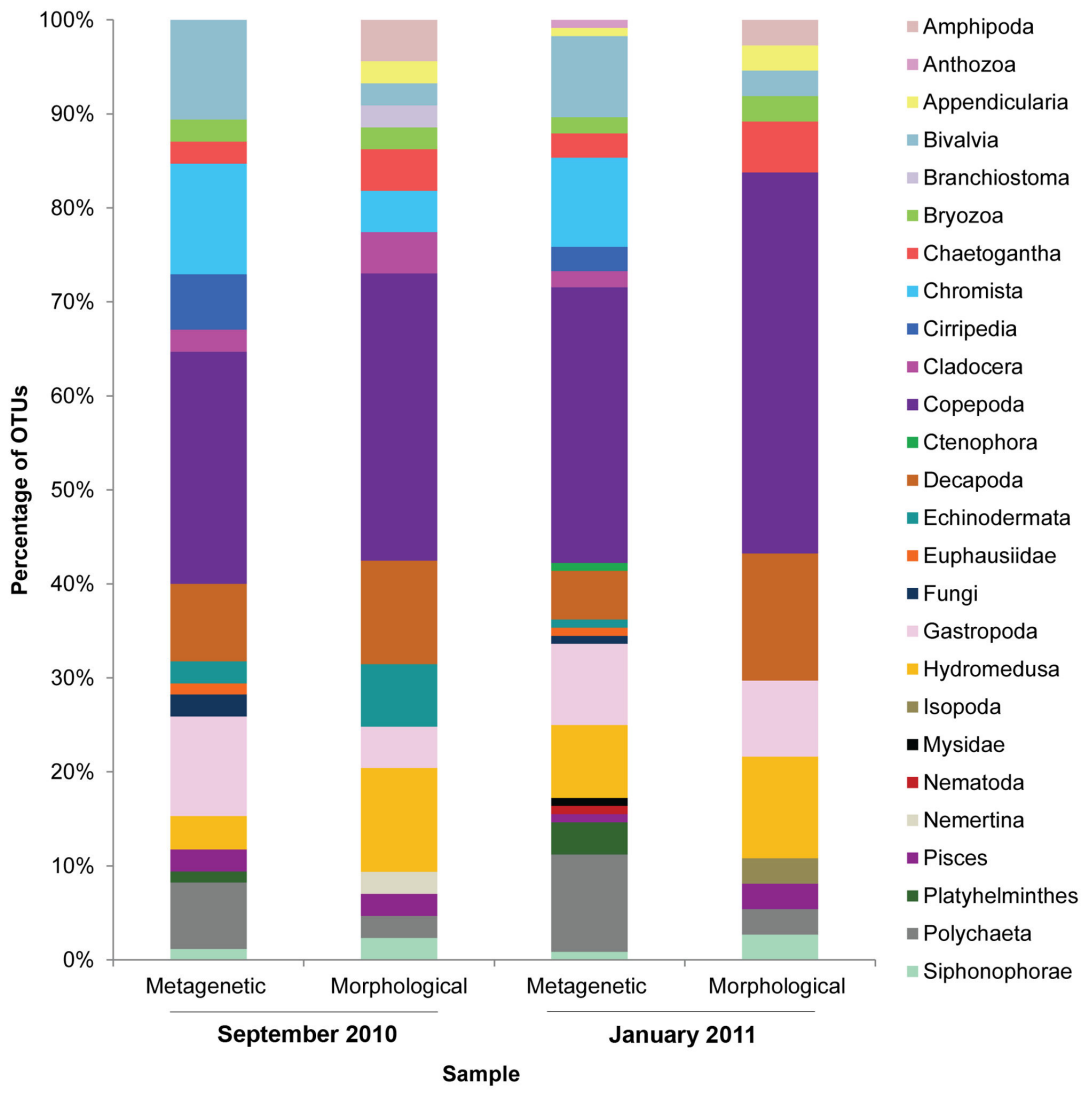
Distribution of OTUs amongst the broad taxonomic groups. Comparison of the number of OTUs within each taxonomic group as a percentage of the total number of OTUs for each analytical method at both time points.

OTUs were put into broad taxonomic groups to allow a comparison between the metagenetic and morphological data. These groups were constrained by the level of identification possible by the morphological analysis. The number of OTUs within each taxonomic group as a percentage of the total OTUs for each method is shown in Figure 2. At this resolution, results determined by the metagenetic analysis broadly align with those determined by morphological analysis. In both September and January the metagenetic and morphological analysis show a diverse range of taxa which in all cases were dominated by Copepoda. 

The number of OTUs generated by both methods is shown in Table 2. In total, metagenetics revealed over three times more OTUs than morphological analysis. The increased taxonomic resolution was predominantly due to the improved identification of meroplanktonic larvae such as Bivalvia, Gastropoda and Polychaeta, as well as some Copepoda that are difficult to identify through microscopy. Metagenetics also detected taxa that are routinely recorded at L4 but were not identified by microscopy in these experimental samples, presumably due to their low abundances, (e.g. Mysida, Cirripedia, Anthozoa). The rarity of these organisms is indicated by the low number of sequences returned for these groups. 

**Table 2:** The number of OTUs generated by metagenetic and morphological analysis.

			**SEPTEMBER 2010**		**JANUARY 2011**			**TOTAL OTUs**		
	**Metagenetic #** OTUs			**Morphological** # OTUs		**Metagenetic**# OTUs	**Morphological** # OTUs		**Metagenetic**	**Morphological**
Amphipoda		0		2		0	1		0	2
Anthozoa		0		0		1	0		1	0
*Appendicularia*		0		1		1	1		1	1
Bivalvia		9		1		10	1		13	1
Branchiostoma		0		1		0	0		0	1
Bryozoa		2		1		2	1		3	1
Chaetognatha		2		2		3	2		3	3
Chromista		10		2		11	0		18	2
Cirripedia		5		0		3	0		6	0
Cladocera		2		2		2	0		2	2
Copepoda		21		14		34	15		40	17
Ctenophora		0		0		1	0		1	0
Decapoda		7		5		6	5		8	8
Echinodermata		2		3		1	0		2	3
Euphausiidae		1		0		1	0		1	0
Fungi		2		0		1	0		2	0
Gastropoda		9		2		10	3		13	4
Hydromedusae		3		5		9	4		9	8
Isopoda		0		0		0	1		0	1
Mysidae		0		0		1	0		1	0
Nematoda		0		0		1	0		1	0
Nermertina		0		1		0	0		0	1
Pisces		2		1		1	1		2	1
Platyhelminthes		1		0		4	0		5	0
Polychaeta		6		1		12	1		14	1
Siphonophorae		1		1		1	1		1	1
Unknowns		26		0		45	0		58	0
**Total OTUs**		**111**		**45**		**161**	**37**		**205**	**58**

Species richness, measured as the average number of recorded species (OTUs), was significantly higher in the metagenetic dataset in both September (t=2.94, p<0.05, d.f.=2) and January (t=4.31, p<0.01, d.f.=2) compared to the morphological dataset.

**Figure 3:**
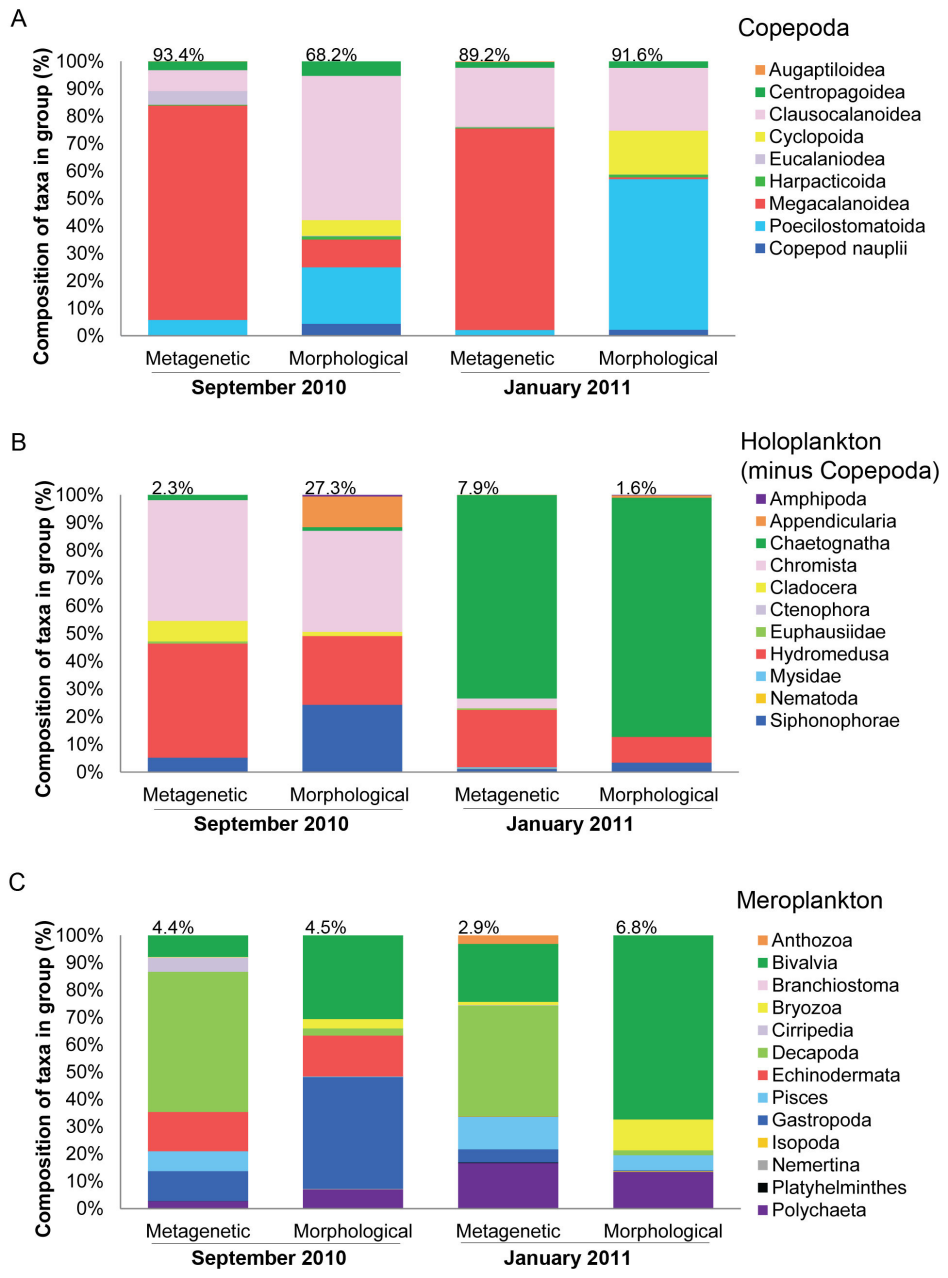
Composition of taxa in the zooplankton samples derived from morphological and metagenetic analysis. The plankton data has been separated into (A) Copepoda, (B) Holoplankton (minus the Copepoda) and (C) Meroplankton. Values above each bar show the percentage contribution of each group to the total number of sequences (metagenetic analysis) or organisms m^-3^ (morphological analysis) for each time point. The bar charts show the composition of taxa present within each group as a percentage of the total number of sequences/organisms in that group.

Both methods showed that copepods strongly dominated in terms of the number of sequences (metagenetic analysis) or abundance of organisms (morphological analysis), ranging from 68% to 93% of the total sequences/organisms for each sample (Figure 3a). However, the relative magnitude of each copepod sub group varies by method and timepoint, as does the composition at genus/species level within each sub group. Morphological analysis showed dominance of the copepods by Clausocalanoidea (mainly unidentified juvenile *Pseudo-/Cteno-/Clausocalanus*) in September and Poecilostomatoida (mainly *Oncaea* spp.) in January. By contrast the Megacalanoidea dominated both metagenetic datasets with 60-70% of their sequences being *Calanus helgolandicus*. Its colder water congener *Calanus finmarchicus* comprised 0.2% of the Megacalanoidea in January and only 0.002% in September. 

While the metagenetics identified more copepod OTUs it failed to identify any Cyclopoid copepods and under-represented the Poecilostomatoida (Figure 3a). *Oithona* and *Oncaea* are common genera at L4 and together made up 21-67% of copepods in the morphological analysis in September and January respectively, but *Oncaea* (Poecilostomatoida) were not identified by metagenetics and only 20 sequences (0.009% of the copepod sequences) were found to be *Oithona similis* (Cyclopoida).

The remaining non-copepod holoplankton comprised 1.6% to 27% of total zooplankton in terms of abundance of sequences/individuals (Figure 3b). In September large numbers of *Noctiluca scintillans* and Hydromedusae in the morphological analysis led to a high proportion of holoplankton (27%) in the total sample. Although Hydromedusae were seen in the metagenetic dataset they comprised only 0.001 % of total sequences, whereas in the morphological dataset they amounted to 8.6 % of the total individuals. In January Chaetognatha dominated the holoplankton in both datasets, with both methods identifying these as Sagittoidea, and morphological analysis also resolving chaetognath eggs and juveniles. 

In September metagenetic and morphological analysis showed an equal percentage of the sequences/individuals to be meroplankton (4.4% and 4.5% respectively), but in January morphological analysis identified 6.8% as meroplankton compared to 2.9% by metagenetics (Figure 3c). The methods reveal domination of the meroplankton by different taxa. Metagenetics showed dominance of Decapoda, in particular *Liocarcinus* spp. at both time points. Morphological analysis showed Gastropoda (41%) and Bivalvia (31%) to dominate the meroplankton in September, while in January Bivalvia remained dominant (67%) and Gastropoda dropped to 0.3%. 

### Taxonomic resolution of the metagenetic and morphological datasets

NGS analysis revealed up to three times more Copepoda OTUs than morphological analysis and allowed identification to species level for all but one OTU. This is often not possible for early copepod stages using morphological analysis, although microscopic examination was able to provide information on life stages and sex of some calanoid copepods. *Calanus helgolandicus*, a common local copepod, was recorded in both September and January, but in January metagenetics also revealed the presence of *Calanus finmarchicus* which was not identified morphologically. 

Meroplanktonic larvae often cannot be identified to species level using morphological techniques alone so there is only one morphological OTU for each of the polychaete, bivalve and gastropod taxonomic groups. The metagenetic analysis however, revealed 14 species of polychaetes, 13 gastropods and 13 bivalve species, thereby improving taxonomic resolution and generating more detailed information about this important group of plankton. 

For both techniques, the Chromista formed a large proportion of the holoplankton in September (Figure 3b). The Chromista and fungi are not within the remit of the zooplankton morphological analysis nor indeed the sampling method, due to their small size. However, the Acantharia and *Noctiluca scintillans*, a large conspicuous dinoflagellate, are readily distinguished using morphological techniques and were recorded by the L4 zooplankton analysts. The presence of high numbers of Chromista and fungi OTUs in the metagenetic dataset probably arose from the guts of zooplankton or adherence to the net, despite not being actively sampled.

Uniquely, the metagenetic analysis of the L4 zooplankton assemblage revealed a number of parasitic species that were not identified in morphological analysis of zooplankton hauls. 9 OTUs were identified as parasites: 2 dinoflagellates, 1 rhizocephalan barnacle, 1 copepod and 5 Platyhelminthes. 

## Discussion

### NGS can be used to estimate taxon richness and diversity of plankton

Advances in NGS technologies have provided the ability to read millions of DNA sequences in parallel, making them ideally suited for large-scale biodiversity analyses of samples [16]. This study has shown that high throughput sequencing of 18S nSSU rRNA gene amplicons can be used successfully to analyse mixed zooplankton assemblages. The success of high throughput sequencing methods depends upon the intended “universality” of the PCR primers used, making the choice of primers pivotal [49]. For this study, primers designed by Fonseca et al. [27] that span the V1-3 region of the 18S nSSU rRNA gene, proved a suitable choice. The locus was beneficial as it has high coverage in public DNA sequence databases, which in conjunction with the amplicon length, enabled better annotation of the returned OTUs.

Application of NGS to a new field raised the question of what depth of sequencing would be needed to represent the majority of zooplankton taxa. We thus ran two technical replicates, one for September and one for January, to determine if the plateau of a species accumulation plot was reached. For the September samples, when the number of reads was highest, the asymptotic value of the curve appears to be about 60,000. Therefore, with the average number of sequences returned for our samples being approximately 63,000, we presume that the sequence coverage was sufficient to represent the majority of OTUs. 

When performing metagenetic assessments of taxon richness, the similarity cut-off used to cluster reads into taxonomic units can result in significantly different estimates of richness [27]. During this study we used the default setting for OTU threshold of 97% recommended for the processing of 18S data in the Qiime pipeline. According to previous studies this level of cut-off may overestimate taxon richness [27]. However, for the analysis of a plankton assemblage, dominated by copepods, we anticipated that a similarity cut-off of 97% would allow taxon richness to be most closely aligned to species richness [50]. Trials showed that for our study if the similarity cut off was reduced from 97% to 96% the OTUs decreased from 205 to 171 (16% decrease) and if this was decreased further to 95% 156 OTUs would be returned (a further 9% decrease). This is a relatively small percentage change compared to previous studies by Fonseca et al. [27] where an increase in similarity cut off from 96% to 97% resulted in a 38% increase of OTUs. In using a similarity cut off of 97% to assemble the reads into OTUs we are therefore not significantly increasing our estimate of taxon richness but are most closely aligning these estimates to species richness.

During this study the suitability of the SILVA database as a reference to assign taxonomy to OTUs was compared with that of the NCBI database. The SILVA database, implemented to provide a comprehensive web resource for quality controlled rRNA sequences, comprises of only high quality, nearly full length sequences [43]. By contrast NCBI consists of an annotated collection of all publically available DNA sequences [51] and is populated by both full and partial length sequences. As such, a comparison with sequences in the NCBI database, including partial sequences submitted from barcoding and phylogenetic studies of copepods, allowed substantially more OTUs to be annotated to species level compared to the SILVA database. This demonstrates that the interpretation of outputs from NGS can be dramatically affected by choice of reference database. 

The annotations were checked against local reference literature [52,53] to ensure that the returned species were local or plausible. Even using NCBI as a reference database we appreciate that the allocation of taxonomy to OTUs may be biased or hindered by a lack of reference sequences. Of the 147 OTUs annotated, 8 were returned as non-local species however, the equivalent local organisms were not in the database. Until the databases are better populated care must be taken with interpretation of these results, especially with respect to determining species which may occur in the ‘rare biosphere’ such as non-indigenous species. From the 17 species that were only recorded in the morphological dataset 14 were not represented in the NCBI database, highlighting the need for effort to be directed at the sequencing of accurately identified organisms in order to populate databases. By the nature of this method we are also reliant on the reference sequences being of high quality and from the unambiguous identification of organisms. 

The number of unassigned OTUs in this study seems high, in total 58 OTUs were classified as unknowns, but these represented less than 2.2% of the total reads. Most of these unassigned OTUs were attributed to the lack of suitable reference sequences present in public databases. By reducing the stringency of the homology threshold the number of unknowns also decreases. In this study decreasing the homology stringency from 97% to 96% resulted in the assignment of a further 5 OTUs (70 sequences), a further reduction to 95% added only another 7 OTUs (63 sequences). None of these additional OTUs belong to a taxon group previously unrepresented or indeed reveal any further rare species. As such, considering the small percentage of sequences that are assigned to unknowns and that a homology threshold of 97% predominantly allows annotation of the OTU to species level we have utilised this stringent threshold of homology.

### A comparison of morphologically and metagenetically derived OTUs assigned to major eukaryotic taxa

Regardless of analysis technique or sampling time, the plankton community was shown to be dominated by Copepoda. This is consistent with the L4 time-series, where copepods represent on average 62% of total zooplankton abundance [5]. 

The analysis of the Copepoda highlights the different values of the two techniques. Metagenetic analysis reveals more copepod OTUs, however, morphological analysis of the Copepoda allows quantification of life stages, such as nauplii and copepodites, and adult males and females, which the metagenetic analysis is not able to do. Early life stages can be difficult to identify any further than “copepod nauplii” or “calanoid juvenile” using microscopy, but all Copepoda OTUs from the metagenetic analysis, except one, could be identified to species level. 

**Figure 4:**
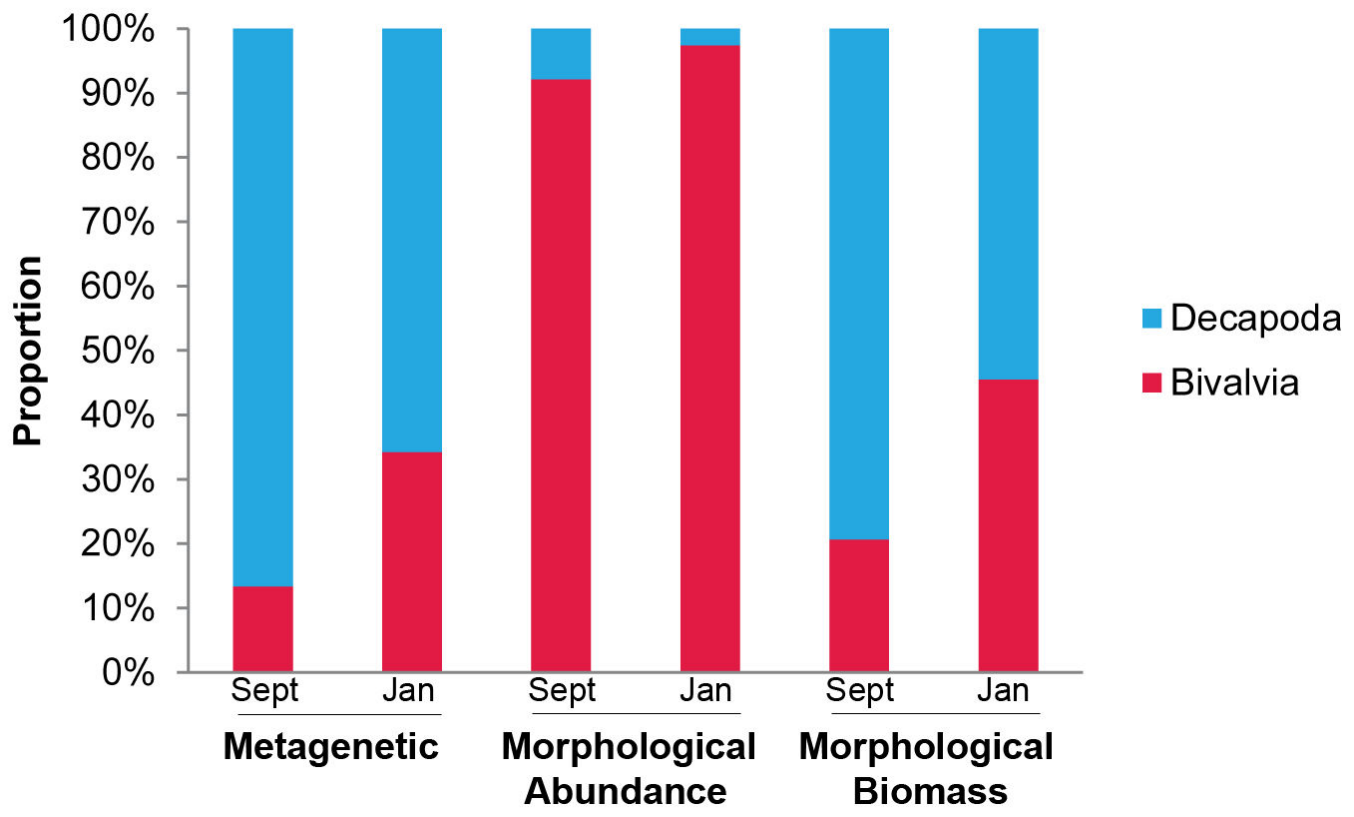
Relative contribution of bivalve and decapod larvae to numbers, biomass and sequence reads. Relative contributions of these larvae at L4, are based on fixed, site-specific conversion factors from abundance to biomass. While the small bivalve larvae are more abundant numerically, the decapods are larger and prevail both in terms of biomass and numbers of sequence reads.

Within the Copepoda the morphological and metagenetic datasets were dominated by different sub-groups; we propose that this is because morphological analysis measured abundance whereas metagenetic analysis is more closely related to biomass. In the case of prokaryotic organisms the number of sequence reads has been used as a surrogate for number of phylotypes, however, the same assumption cannot be made when sequencing multicellular organisms [54]. Multi-copy genes, such as ribosomal genes, vary in copy number across different animals [30,55]. The proportion of DNA sequences generated is affected by the biomass of the different plankton, but the relationship between biomass and sequence generation is not linear and is affected by biases, which can be introduced at a number of different stages such as DNA extraction, PCR, DNA pooling and bioinformatic sorting [56]. We cannot test the strength of the relationship between number of sequence reads and biomass for our whole dataset because we have not yet got biomass conversion factors for the plethora of species and maturity stages we encounter at L4. However L4-specific conversion factors for decapod and bivalve larvae do exist (Figure 4), and these support our suggestion that the number of sequence reads relates better to biomass than numbers. This would also explain the fact that the Copepoda sequences were dominated by *Calanus helgolandicus*, widely reported to form a predominant proportion of the zooplankton biomass in the North Atlantic [5,8]. *Calanus helgolandicus* have a relatively large biomass and are therefore likely to result in a high number of sequence reads. Conversely, morphological analysis showed Copepoda to be dominated in September by small juvenile stages of *Pseudo-/Cteno-/Clausocalanus*, which are high in abundance relative to biomass. 

Variations between datasets may also be attributable to the lack of annotation of the metagenetically derived unknowns. For example, according to morphological analysis, in January the Copepoda were dominated by *Oncaea*. However, the NCBI database is poorly populated with any substantial length reference sequences for *Oncaea* and therefore any such reads would fall into the unknown category. *Acartia clausi*, a copepod commonly recorded at L4 by morphological analysis, is not represented on the database at all. While *Oithona similis* was picked up by metagenetic analysis in January, the number of sequences was low and represented only 0.009% of copepod sequences. This under-representation may have been due to the fact only half of the possible local species of *Oithona* are in the database.

A third source of discrepancy between datasets may result from primer mismatch. Our data has been strictly quality controlled in the Qiime pipeline, and has a zero tolerance for primer mismatch. The primers used in this study are reported to provide reduced amplification of Cnidarian DNA due to a base pair mismatch in the penultimate 3’ position of the reverse primer [25], present in approximately 50 % of all Cnidaria. This mismatch may mean that the primer is unable to bind stably and the DNA is not amplified, it is also possible that some good quality Cnidaria sequences will not have made it through the de-noiser step in Qiime due to primer base mismatch. To test this, the DNA sequences were reprocessed by altering Qiime’s primer mismatch parameters. However, altering this parameter did not alter the number of OTUs that were returned, only the number of sequences written, until the level of mismatch in the primers reached 7. The lack of generation of new OTUs until the primer mismatch reached such extreme levels implies that there are other reasons for the under-representation of Cnidaria in terms of the number of sequences returned by metagenetics compared to the morphological outputs. The low sequence reads returned for Cnidaria could potentially be due to the low DNA content compared to biomass within this group. 

### Metagenetics reveals hidden taxon richness

Metagenetic analysis revealed greater species richness than was possible with morphological identification. This reflects the identification of organisms that were not detected by morphology such as internal parasites, increased resolution within groups that cannot easily be identified to species level by microscopy or those that were too low in abundance to be observed by microscopy. Increased resolution was particularly evident in meroplankton and the Copepoda. Metagenetics revealed the presence of *Calanus finmarchicus*, a cold-temperate water species, which despite co-occurring with *Calanus helgolandicus* in many areas of the North Atlantic is relatively rare at L4. The early copepodite stages of these species are not possible to distinguish morphologically, and either for this reason or because of their great rarity *C. finmarchicus* was not recorded by morphological analysis. This highlights the advantages of NGS in detecting rare or juvenile species. Correct identification of the *C. finmarchicus* and *C. helgolandicus* pair is important as the relative abundance of these species has been widely used as an indicator of climatic changes [57] and their differing life cycles have major influences on the marine food web [58]. 

Although meroplankton accounted for only 2.9%-6.8% of total individuals/reads in the spring/summer, when benthic species reproduce most actively the numerical contribution of meroplankton can reach 43% [36]. Morphological analysis allowed allocation of organisms to broad taxonomic level, but metagenetic analysis provided greater resolution to these taxa, with OTUs identified to species or genus level. Within the Bivalvia, 13 OTUs were returned from metagenetic analysis and assigned to a number of species recorded at L4 such as *Phaxus pellucidus, Abra alba* and *Musculus* spp. [52]. Two species of burrowing piddock were also recorded, which are found in algal holdfasts or burrowing into soft rock and clay. L4 has a sandy seabed, which implies that larvae of these species must be advected by currents to L4. Metagenetic analysis also improved taxonomic resolution of the Polychaeta and Gastropoda. High throughput sequencing therefore reveals a previously hidden taxon richness of these non-permanent planktonic animals in their dispersal phase. Detailed descriptions of larval abundance, impossible to provide based on morphological observations alone, can help to understand important aspects of pelagic ecosystems and bentho-pelagic coupling such as functional diversity, dispersal and time of spawning.

Some taxa that are routinely recorded at L4 were detected by NGS but not by microscopy. Cirripedia, Ctenophora and Anthozoa can all reach high numbers at certain times of the year, and a small number of sequences were detected for each of these groups, even though none were recorded during microscopic analysis. These organisms may have been overlooked for a number of reasons, most likely their extremely low abundance in samples, making them difficult to detect in the large subsample which can contain very high numbers of organisms. Alternatively, these DNA sequences may have arisen from fragments of organisms, eggs, or the gut contents of other zooplankton, which would not have been recorded by microscopists. 

Metagenetic analysis revealed further diversity within the zooplankton community in the form of parasites. All zooplanktonic organisms are hosts to parasites ranging from viruses and bacteria to Metazoa [59]. Metagenetic analysis returned two parasitic dinoflagellates that infect copepods, *Blastodinium navicula* and *Syndinium turbo*, both of which affect the copepod life cycle and can result in mortality [60,61]. A meroplanktonic rhizocephalan, *Peltogaster paguri*, which causes sterility in pagurid crabs [62,63] and the non-pathogenic parasitic copepod, *Taeniacanthus zeugopteri*, found on flatfish [64] were also revealed by the metagenetic analysis. The pathogenic parasites revealed by NGS may influence zooplankton populations and fluctuations in abundance. 

### Perspectives

Large-scale patterns of diversity have long interested and puzzled ecologists [65]. However, the quantification of global-scale patterns in diversity is made problematic when using microscopy due to the difficulty of identifying rare species and larval stages of meroplankton and copepods. There have been a variety of descriptions of latitudinal and shelf-oceanic gradients in zooplankton diversity based on microscopy [4,66,67]. These often concentrate only on adult copepods grouped at the genus level to reduce the risk of identification inconsistencies at the species level. We have shown that when analysed by a trained microscopist, analysis of the zooplankton community may display only about 50% of its “real” copepod diversity as revealed by NGS. These problems are fundamental and often taken for granted, but may lead to severe underestimations of global zooplankton diversity and misrepresentation of clines in diversity.

Metagenetic analysis of global species richness based on NGS will provide a measurement of how many OTUs there are within major groups in a given volume of water. If methods can be standardised then results will be comparable and will give a good indication of biological patterns, macro diversity and habitat-species richness, regardless of how well annotated the OTUs are. In fact one benefit of NGS is that providing methods are correctly recorded raw sequence data will remain available for future analysis allowing a comparison of diversity from multiple studies.

 The benefit of NGS in providing a broad assessment of diversity and greater resolution of taxon richness is evident but we should not oversell these technologies in their current state, or pretend that they replace the detailed visual examination of the samples, as each approach has strengths and weaknesses (Table 3). An often-forgotten element of zooplankton research is the enormous requirement of time and skill from laboratory analysts to process the samples. The increasing demand for quality-controlled time series linking ocean observatories into networks [68] is concomitant with an increasing rarity of skilled taxonomists [69]. The high throughput 18S nSSU rDNA amplicon sequencing approach of this study may become increasingly attractive if there are on-going trends of a) non-replacement of retiring taxonomists, b) reducing costs and automation of molecular approaches and c) improvement of molecular reference libraries. The need for expert taxonomists is evident if we consider this last point. Regardless of how many sequences we can produce simultaneously at a low cost they are of most value if they can be reliably annotated against a well-populated reference library that is based on correctly identified specimens. The need for such a library should not be underestimated and a combined morphological and molecular approach to provide accurate sequences of correctly identified specimens should be prioritised in future research. 

**Table 3:** Comparison of the Metagenetic and Morphological analytical methods.

**Outputs**	**Metagenetic**	**Morphological**
Unit of identification	Number of readable sequences (crudely approximates to biomass)	Usually numbers, conversions to biomass possible
Identification of rare species	Possible, given presence in database	Time consuming
Identification of morphologically similar organisms (e.g. some meroplankton, most larval stages)	Possible	Very difficult and time consuming
Identification of individual life stages	Not possible	Possible for some species
Standardisation of approach across different labs	Possible	Difficult, requires repeated testing and QC
